# Optimizing Cardiomyocyte Differentiation: Comparative Analysis of Bone Marrow and Adipose-Derived Mesenchymal Stem Cells in Rats Using 5-Azacytidine and Low-Dose FGF and IGF Treatment

**DOI:** 10.3390/biomedicines12081923

**Published:** 2024-08-22

**Authors:** Ahmed Farag, Sai Koung Ngeun, Masahiro Kaneda, Mohamed Aboubakr, Ryou Tanaka

**Affiliations:** 1 Veterinary Teaching Hospital, Faculty of Agriculture, Tokyo University of Agriculture and Technology, Tokyo 183-8509, Japan; 2 Department of Surgery, Anesthesiology, and Radiology, Faculty of Veterinary Medicine, Zagazig University, Zagazig 44519, Egypt; 3 Laboratory of Veterinary Diagnostic Imaging, Faculty of Agriculture, Tokyo University of Agriculture and Technology, Tokyo 183-8509, Japan; s212892q@st.go.tuat.ac.jp; 4 Laboratory of Veterinary Anatomy, Division of Animal Life Science, Tokyo University of Agriculture and Technology, Tokyo 183-8509, Japan; kanedam@cc.tuat.ac.jp; 5 Department of Pharmacology, Faculty of Veterinary Medicine, Benha University, Toukh 13736, Egypt; mohamedhafez19@yahoo.com

**Keywords:** Mesenchymal stem cells, cardiomyogenic differentiation, 5-Azacytidine, growth factors

## Abstract

Mesenchymal stem cells (MSCs) exhibit multipotency, self-renewal, and immune-modulatory properties, making them promising in regenerative medicine, particularly in cardiovascular treatments. However, optimizing the MSC source and induction method of cardiac differentiation is challenging. This study compares the cardiomyogenic potential of bone marrow (BM)-MSCs and adipose-derived (AD)-MSCs using 5-Azacytidine (5-Aza) alone or combined with low doses of Fibroblast Growth Factor (FGF) and Insulin-like Growth Factor (IGF). BM-MSCs and AD-MSCs were differentiated using two protocols: 10 μmol 5-Aza alone and 10 μmol 5-Aza with 1 ng/mL FGF and 10 ng/mL IGF. Morphological, transcriptional, and translational analyses, along with cell viability assessments, were performed. Both the MSC types exhibited similar morphological changes; however, AD-MSCs achieved 70–80% confluence faster than BM-MSCs. Surface marker profiling confirmed CD29 and CD90 positivity and CD45 negativity. The differentiation protocols led to cell flattening and myotube formation, with earlier differentiation in AD-MSCs. The combined protocol reduced cell mortality in BM-MSCs and enhanced the expression of cardiac markers (*MEF2c*, *Troponin I*, *GSK-3β*), particularly in BM-MSCs. Immunofluorescence confirmed cardiac-specific protein expression in all the treated groups. Both MSC types exhibited the expression of cardiac-specific markers indicative of cardiomyogenic differentiation, with the combined treatment showing superior efficiency for BM-MSCs.

## 1. Introduction

Cardiovascular diseases (CVDs) are the foremost cause of mortality worldwide, primarily resulting from the irreversible loss of functional cardiomyocytes [1,2,3]. Over the years, a wide range of therapeutic modalities and materials have been employed to address this critical issue [4,5]. Among the promising therapeutic strategies, stem cell-based treatments, especially those utilizing mesenchymal stem cells (MSCs), are gaining attention for their potential to regenerate the myocardium and enhance cardiac function following myocardial infarction (MI) [6,7]. Over the past decades, MSCs have been extensively researched for their capability to differentiate into various cell lineages, positioning them as a promising candidate in cardiac regenerative therapy. Their high plasticity, secretory profile, and immunomodulatory properties further underscore their potential in effectively regenerating the damaged myocardium [8,9,10].

MSCs are abundantly accessible from multiple sources, including bone marrow. Bone marrow-derived mesenchymal stem cells are highly valued in cellular therapy because of their immunological neutrality, self-renewal capability, and multipotent differentiation potential [11,12]. Additionally, adipose tissue serves as a viable source of stem cells, given its widespread availability and ease of extraction. MSCs isolated from adipose tissue exhibit robust proliferation and the capability to differentiate into functional cardiac lineage cells [13,14]. Both bone marrow-derived and adipose tissue-derived MSCs exhibit comparable morphological, phenotypic, and immunosuppressive characteristics [15]. Nevertheless, adipose tissue stands out for its practical advantages, including easier accessibility, simpler isolation methods, and greater proliferative potential compared with bone marrow. The minimally invasive nature of adipose tissue harvesting is noteworthy, and its widespread distribution throughout the body enables collection from multiple sites. This approach facilitates the extraction of large quantities of cells in a single procedure, eliminating the need for extensive in vitro expansion [16].

Frequently employed chemical stimuli, such as 5-Aza, oxytocin, trichostatin A (TSA), 3-deazaneplanocin A (DZNep), and dimethyl sulfoxide (DMSO), are utilized to induce cardiac differentiation in vitro [17]. 5-Aza is commonly utilized to induce cardiac differentiation in MSCs by inhibiting DNA methyltransferase, facilitating DNA demethylation, and promoting the expression of cardiac genes [18]. However, recent studies have highlighted the inadequacy of 5-Aza as a biochemical inducer of cardiomyogenic expression in adipose-derived MSCs, citing its cytotoxic effects [19,20]. Consequently, researchers are exploring alternative methods to direct MSC differentiation towards cardiomyocytes. Growth factors are peptide molecules with multifaceted roles in controlling essential biological functions like cell signaling, proliferation, survival, and differentiation. In living organisms, cells release these growth factors in response to injury, effectively stimulating and regulating the proliferation and differentiation of local stem cell populations [21]. Basic fibroblast growth factor (bFGF) and insulin-like growth factor-1 (IGF-1) are among the growth factors commonly employed in differentiation protocols. bFGF is particularly critical during early embryonic development, influencing mesodermal lineage commitment during cardiac organogenesis [22,23]. IGF-1, renowned for its cardioprotective effects, modulates cell proliferation and inhibits apoptosis in cardiomyocytes post-myocardial infarction [24].

We used rats as the model animals in our study to explore the cardiomyogenic potential of MSCs, due to their similarities and specific advantages compared with human MSCs (hMSCs). Rat MSCs (rMSCs) share comparable subpopulations and respond well to low-density plating, enhancing expansion. They also exhibit unique traits, such as a greater sensitivity to plating density, rapid expansion, frequent medium requirements, and the ability to form confluent cultures and generate single-cell-derived colonies [25]. These characteristics make rMSCs an effective and responsive model for evaluating cardiomyocyte generation protocols, providing insights that are applicable to human treatments.

In this study, we examined and compared the cardiomyogenic potential of BM-MSCs and AD-MSCs when treated with 5-Azacytidine alone and in combination with low doses of fibroblast growth factor and insulin-like growth factor. Our aim is to identify the most effective cell source and induction method for potential future in vivo transplantation applications.

## 2. Materials and Methods

### 2.1. Study Design

**Figure 1:**
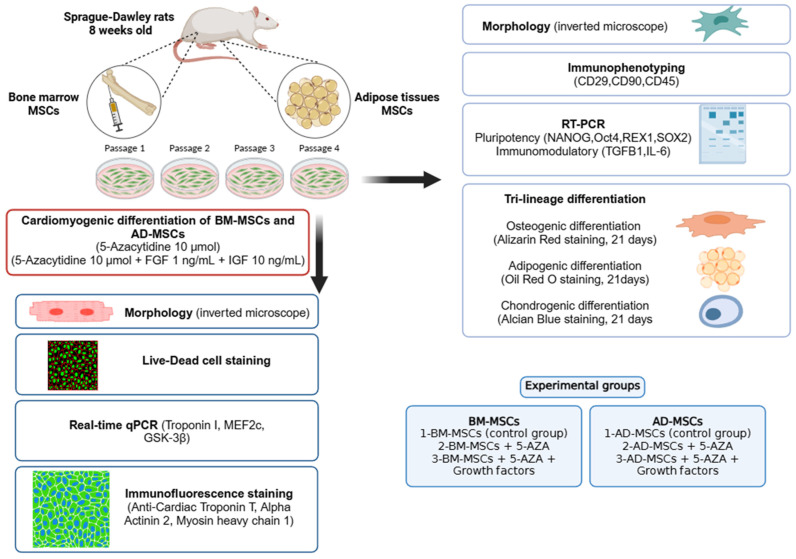
Experimental design. BM-MSCs, bone marrow mesenchymal stem cells; AD-MSCs, adipose tissue mesenchymal stem cells; AZA, 5-Azacytidine.

The study was carried out in accordance with the protocols approved by the Animal Care and Use Committee of Tokyo University of Agriculture and Technology (approval no: R05-158). Figure 1 outlines the general framework of the experimental procedures.

### 2.2. Isolation of MSCs from Bone Marrow

The femurs and tibiae of four eight-week-old male Sprague–Dawley rats were harvested following euthanasia. The procedure involved pulling the skin towards the feet and severing it at the ankle bone, followed by the meticulous removal of muscle and connective tissue from both bones. This was achieved by cleaning the shafts and pulling the tissue towards the ends of the bones. The bones were sterilized in 10% ethyl alcohol for a few seconds (this step is effective in avoiding contamination during the isolation procedures). Sharp scissors were used to cut the ends of the tibiae and femurs, after which a 27-gauge needle was inserted and flushed with Dulbecco’s Modified Eagle’s Medium (DMEM) (cat. no. 043-30085, FUJIFILM Wako Pure Chemical Corporation, Osaka, Japan), which was collected in a 15-mL tube. To purify the bone marrow samples and eliminate unwanted blood cells, an RBC lysis protocol (cat. no. 00-4333-57, eBioscience™ Promega, Mannheim, Germany) was employed. After storing the samples at 4 °C for 10 min, they underwent centrifugation at 300× *g* for 10 min to remove the supernatant. The resulting cell pellets were rinsed with phosphate-buffered saline (PBS) (cat. no. 09-8912-100, Medicago AB, Uppsala, Sweden). Finally, the cell pellets were plated in a basal culture medium composed of DMEM supplemented with 10% fetal bovine serum (FBS) (cat. no. CCP-FBS-BR-500, COSMOBIO, Tokyo, Japan), 1% non-essential amino acids (cat. no. 139-15651, FUJIFILM Wako Pure Chemical Corporation, Osaka, Japan), and 1% penicillin/streptomycin (cat. no. 161-23181, FUJIFILM Wako Pure Chemical Corporation, Osaka, Japan) [26].

### 2.3. Isolation of MSCs from Adipose Tissue

The inguinal fat pads from four eight-week-old male Sprague–Dawley rats were collected under sterile conditions. These adipose tissues were placed in a tube containing PBS and transported to the laboratory. Once there, the samples were rinsed with PBS. In a biosafety cabinet, the tissues were finely chopped with sterile scissors in a 60-mm diameter culture dish (cat. no. TR4001, Nippon Genetics Co., Ltd., Tokyo, Japan). The minced tissues were subsequently placed in a shaking water bath at 37 °C and gently agitated for 1 h. The water bath contained Hank’s balanced salt solution (HBSS) (cat. no. 14025-092, Thermo Fisher Scientific Inc., New York, NY, USA) with an addition of 0.1% collagenase type 1 (cat. no. 17100017, Gibco by Life Technologies, Waltham, MA, USA). To neutralize the collagenase activity, DMEM supplemented with 10% FBS was added. Large cell aggregates were then eliminated using a 100 μm filter (BD Falcon, Bedford, MA, USA). The cells were subsequently centrifuged at 800× *g* for 10 min, after which the supernatant was discarded. The resulting cell pellets were resuspended in 1 mL of an RBC lysis buffer to lyse any remaining red blood cells. After incubating with the RBC lysis buffer for 10 min at 4 °C, the cells underwent a washing step with 10 mL of PBS. Subsequently, they were centrifuged again at 600× *g* for 3 min, and the supernatant was removed. The cell pellets were then resuspended in DMEM supplemented with 10% FBS, 1% non-essential amino acids, and 1% penicillin/streptomycin, which served as the basal culture medium [27].

### 2.4. Cell Cultivation

Maintaining optimal conditions for culturing MSCs is crucial for their survival, differentiation, and proliferation. This includes using a suitable growth medium, and maintaining an appropriate temperature, gas pressure, and humidity. The MSCs were cultured in a 100-mm diameter culture dish (cat. no. TR4002, Nippon Genetics Co., Ltd., Tokyo, Japan) and placed in an incubator set at 37 °C with 5% CO_2_ in a humidified environment. Upon reaching 80% confluency, the cells were subcultured at a split ratio of 1:2. This cycle was repeated until passage 4.

### 2.5. Morphology and Immunophenotyping

The cell morphology was evaluated using an inverted microscope (Olympus CKX31, Tokyo, Japan). At passage 4, MSCs from both sources underwent a flow cytometry analysis to identify the standard MSC surface markers. The antibodies used were CD29 (1 µg/test, PE, eBioscience™, cat. no. 12-0291-82) and CD90 (0.06 µg/test, PE, eBioscience™, cat. no. 12-0900-81) for the MSC surface markers, and CD45 (0.5 µg/1 × 10^6^ cells, PE, cat. no. MA5-17379) as a hematopoietic cell marker antibody. The isotype controls included the Armenian Hamster IgG Isotype Control (0.25 µg/test, PE, eBioscience™, cat. no. 12-4888-81), the Mouse IgG2a kappa Isotype Control (0.5 µg/test, PE, eBioscience™, cat. no. 12-4724-42), and the Mouse IgG2a Isotype Control (0.4 µg/test, PE, cat. no. MG2A04).

Before the flow cytometry analysis, cells were washed three times with HBSS and adjusted to a concentration of 1 × 10^6^ cells/mL. Subsequently, cell suspensions were incubated in the dark at 4 °C for 20 min with the appropriate antibodies, using the concentrations recommended by the manufacturer. After incubation, cells were washed with HBSS to remove any unbound antibodies. A flow cytometry analysis to detect the cell surface antigens and determine their expression percentages was carried out using a Beckman Coulter flow cytometer (Brea, CA, USA). The data analysis was performed using the CytExpert Software version 2.3 (Beckman Coulter, Brea, CA, USA).

### 2.6. Tri-Lineage Differentiation

Plastic-adherent MSCs at the fourth passage underwent differentiation using specific induction media to evaluate their adipogenic, chondrogenic, and osteogenic potential. Undifferentiated BM-MSCs and AD-MSCs cultured in a standard medium were used as the negative controls. Tissue-specific staining rates were assessed using ImageJ (1.8.0-345) (https://imagej.nih.gov/ij/download.html (accessed on 10 July 2024)). The analysis involved determining the percentage of stained cells within the observation field for each sample.

#### 2.6.1. Adipogenic Differentiation

The cells were seeded into each well of a 6-well plate at a density of 1 × 10^5^ cells per well (cat. no. TR5000, Nippon Genetics Co., Tokyo, Japan). Upon reaching 80% confluence, adipogenic differentiation was initiated with DMEM supplemented with 10% FBS, along with 1 μM dexamethasone (cat. no. D4902, Sigma Aldrich, St. Louis, MO, USA), 500 μM isobutylmethylxanthine (cat. no. I5879-100MG, Sigma Aldrich, St. Louis, MO, USA), 100 μM indomethacin (cat. no. I7378-5G, Sigma Aldrich, St. Louis, MO, USA), and 5 μg/mL insulin (cat. no. I5500-50MG, Sigma Aldrich, St. Louis, MO, USA). The medium was refreshed every 3 days throughout a 21-day period of exposure to the adipogenic induction medium. After this duration, Oil Red O staining was performed on the cells. Initially, the cells were washed with PBS and then fixed with 4% paraformaldehyde for 15 min. Subsequently, the samples were stained with 0.5% Oil Red O (cat. no. O-0625, Sigma-Aldrich, St. Louis, MO, USA) dissolved in a 3:2 mixture of isopropanol and distilled water for 10 min. This was followed by two washes with distilled water (dH_2_O). This staining process was used to detect fat vacuole formation within the cells. The presence of red-stained fat vacuoles confirmed the successful differentiation into adipocytes.

#### 2.6.2. Chondrogenic Differentiation

The cells were plated in each well of a 6-well plate at a density of 1 × 10^5^ cells per well. Chondrogenic differentiation was induced upon reaching 80% confluence. A commercially available kit, specifically a serum-free cell culture medium (cat. no. C-28012, Promo Cell, Heidelberg, Germany), was employed to promote chondrogenic lineage development. The medium was refreshed every 3 days throughout a 21-day period. Following the 21-day period of chondrogenic induction, Alcian Blue staining was conducted. The cells were washed twice with PBS and subsequently fixed with 4% paraformaldehyde for 30 min at room temperature. After the fixation, the cells were rinsed with distilled water and stained overnight at room temperature in the dark with 1% Alcian Blue (cat. no. 66011-100ML F, Sigma-Aldrich, St. Louis, MO, USA). On the next day, the staining solution was aspirated, and the cells were washed three times with 0.1 N hydrochloric acid (HCl) (cat. no. 083-01115, FUJIFILM Wako Pure Chemical Corporation, Osaka, Japan). This staining procedure was employed to assess chondrocyte formation, emphasizing the presence of highly sulfated proteoglycans within the cartilage matrix.

#### 2.6.3. Osteogenic Differentiation

Likewise, in a 6-well plate, the cells were seeded at a density of 1 × 10^5^ cells per well. Upon reaching 80% confluence, the cells were subjected to osteogenic differentiation by introducing a medium composed of DMEM supplemented with 10% FBS, 100 nM dexamethasone, 0.2 mM ascorbic acid (cat. no. 016-04805, FUJIFILM Wako Pure Chemical Corporation, Osaka, Japan), and 10 mM β-glycerophosphate (cat. no. G9422-10G, Sigma Aldrich, St. Louis, MO, USA). The medium was changed every 3 days for a period of 21 days. Following 21 days of osteogenic induction, Alizarin Red (ALZ) staining was performed. The cells were washed twice with PBS and then fixed in ice-chilled 70% ethanol for 1 h at 4 °C, followed by two rinses with distilled water. Next, the ALZ solution (cat. no. 40-1009-5, Sigma-Aldrich, St. Louis, MO, USA) was applied to completely cover the cells and incubated for 30 min at room temperature. Subsequently, the wells were washed twice with distilled water. The appearance of red staining in the matrix indicated successful osteogenic differentiation.

### 2.7. Gene Expression of MSCs by Reverse-Transcriptase PCR

Reverse-transcription PCR (RT-PCR) was conducted to evaluate the expression of pluripotent and immunomodulatory genes. Pluripotent markers, such as embryonic stem cell-specific homeobox (*NANOG*), Octamer-binding transcription factor 4 (*Oct4*), SRY-box containing gene 2 (*SOX2*), and reduced expression1 (*REX1*), along with immunomodulatory markers, including transforming growth factor beta 1 (*TGFB1*) and interleukin 6 (*IL-6*), were assessed. To ensure PCR efficacy, β-actin was utilized as a control.

Rat BM-MSCs and AD-MSCs at the fourth passage were used to isolate the total RNA using the FastGene RNA Premium Kit (NIPPON Genetics, Tokyo, Japan). RNA concentration and quality were assessed using a NanoDrop 2000 ultra-micro spectrophotometer (Thermo Fisher Scientific, Cambridge, MA, USA). Subsequently, 1 μg of RNA with an OD_260_/OD_280_ ratio of 2.0 was reverse transcribed using the PrimeScript RT Reagent Kit (Takara Bio, Shiga, Japan). The resulting cDNA was utilized in PCR amplification, with the PCR cycling performed using a Veriti Thermal Cycler (Thermo Fisher Scientific, Waltham, MA, USA), following the established protocols [28]. The specific primer details are provided in Table 1.

### 2.8. Cardiomyogenic Differentiation of BM-MSCs and AD-MSCs

BM-MSCs and AD-MSCs at the fourth passage were treated with 10 μmol of 5-Azacytidine (cat. no. A2385, Sigma-Aldrich) for 24 h [29,30]. Following treatment, they underwent two washes with 1 × PBS and were then cultured in basal media comprising DMEM and 10% FBS, designated as the (BM-MSCs + 5 Aza and AD-MSCs + 5 Aza) groups. Concurrently, other cultures were exposed to a combination of 10 μmol of 5-Azacytidine along with low doses of growth factors, specifically 1 ng/mL FGF (cat. no. 10018B, PeproTech, Cranbury, NJ, USA) [31] and 10 ng/mL IGF (cat. no. 100-11, PeproTech, Cranbury, NJ, USA) [32,33]. After 24 h of exposure to this mixture, the BM-MSCs and AD-MSCs underwent two washes with PBS and were subsequently maintained with the growth factors alone in the basal media, referred to as the (BM-MSCs + 5 Aza + GFs and AD-MSCs + 5 Aza + GFs) groups. The BM-MSCs and AD-MSCs cultured solely in the basal media without any treatment served as the assay controls.

### 2.9. Morphological Analysis after Differentiation

Throughout the 3-week induction period, the cells were monitored daily for changes in morphology, such as cell flattening, multinucleation, and the formation of myotube-like structures. The observations of these alterations were documented, and images were taken using an inverted microscope at the end of the induction period.

### 2.10. Live/Dead Cell Staining

At 7 days post-induction, the cell viability in the experimental groups was evaluated. The cells were subjected to a 15-min incubation at 37 °C using the Live/Dead Cell Viability Assay Kit (cat. no. ab287858) according to the manufacturer’s instructions. Following the incubation, the cells were promptly examined under a fluorescence microscope (BZ-9000, KEYENCE Instruments Inc., Tokyo, Japan). The healthy cells exhibited a green stain with the Cell Dye II/Live Cell Staining Dye, while the dead cells displayed a red stain [34]. The Live/Dead assay provided a direct assessment of the proportion of living and dead cells using a two-color fluorescence method. This technique employs two different dyes: one penetrates the intact, live cells and is hydrolyzed by intracellular esterases, producing a green, fluorescent compound that remains in the cell cytoplasm; the other enters the cells through damaged membranes, binds to nucleic acids, and emits a bright red fluorescence in the dead cells. The images were analyzed using ImageJ (available at https://imagej.nih.gov/ij/download.html), where the cells were counted. The cell viability was calculated as the percentage of green cells relative to the total number of cells.

### 2.11. Quantitative Assessment of the Cardiac-Specific Genes Using RT-qPCR

The total RNA from both the BM-MSCs and AD-MSCs in the control and treatment groups was extracted using the FastGene RNA Premium Kit (Nippon Genetics, Tokyo, Japan), following the manufacturer’s instructions. The concentration and purity of RNA (OD_260_/OD_280_ ≈ 2.0) were assessed using a NanoDrop 2000 ultra-micro spectrophotometer (Thermo Fisher Scientific, Cambridge, MA, USA). Subsequently, 1 µg of RNA was utilized for cDNA synthesis using the PrimeScript RT Reagent Kit (Takara Bio, Shiga, Japan).

The expression levels of various multipotent genes, including the cardiomyocyte-specific gene Myocyte enhancer factor 2C (*MEF2c*) and cardiac-specific markers, such as *Troponin I* and Glycogen synthase kinase-3 beta (*GSK-3β*), were quantified utilizing the StepOnePlus™ Real-Time PCR System (Thermo Fisher Scientific, Waltham, MA, USA). The qPCR reaction mixture was prepared as follows: 1 µL of cDNA, 0.5 µL of each forward and reverse primer (both at 10 µmol/L), 10 µL of THUNDERBIRD^®^ Next SYBR^®^ qPCR Mix (Toyobo Life Science, Osaka, Japan), and 8 µL of ddH_2_O. The 2^−∆∆^Cq method was employed for relative quantification, with normalization to β-actin [35]. The primers that were used are specified in Table 2.

### 2.12. Immunofluorescence Staining of Cardiomyocyte-Specific Proteins

Three weeks after the induction, immunofluorescence staining was performed to evaluate the cardiomyocyte differentiation of the MSCs in the treated experimental groups. This assessment targeted the cardiomyocyte-specific proteins, Anti-Cardiac Troponin T (cTnT), alpha Actinin 2 (α-Actinin), and Myosin heavy chain 1 (MHC).

Briefly, the culture medium was aspirated, and the cells were gently rinsed once with PBS. After aspirating the PBS, the cells were fixed with 4% paraformaldehyde (cat. no. 09154-85) at 4 °C for 15 min. The fixative was then aspirated, and the cells were rinsed once with PBS. Next, the cells were permeabilized with 0.5% Triton X-100 (cat. no. 9036-19-5) for 10 min. After aspirating the Triton X-100, the cells were rinsed once with PBS and then blocked with 1% bovine serum albumin (BSA) (cat. no. A9418-5G) for 30 min at room temperature.

Following the removal of BSA, the cells were incubated with cardiac-specific primary antibodies for 1 h at 20 °C. The antibodies used were cTnT (cat. no. ab209813; 1:400; Abcam, Cambridge, UK) [36], α-Actinin (cat. no. 701914, 1:100, Invitrogen, Waltham, MA, USA) [37], and MHC (cat. no. PA5-117077, 1:200, Invitrogen, Waltham, MA, USA) [38]. After the incubation period, the primary antibodies were removed by washing the cells twice with PBS, followed by incubation with the secondary antibodies (Goat Anti-Rabbit IgG H&L (FITC), 1:1000, ab6717, Abcam, and Goat anti-Rabbit IgG (H+L) Cross-Adsorbed Secondary Antibody, FITC, 2 µg/mL, F-2765, Invitrogen) for 1 h at room temperature, protected from direct light.

The cell nuclei were then counterstained with 4′,6-diamidino-2-phenylindole (DAPI, 5 μg/mL; 1:1000, Catalog No. D3571, Invitrogen) [39] for 5 min. The resulting slides were studied using a fluorescence microscope BZ-9000 (KEYENCE Instruments Inc., Tokyo, Japan).

### 2.13. Statistical Analysis

Mean ± standard deviation values were used to present all the data. A student’s *t*-test was used to compare the continuous variables between BM-MSCs and AD-MSCs. A one-way ANOVA was employed for multiple group comparisons. Statistical significance was defined as *p* < 0.05. The data analysis was performed using GraphPad Prism 8.0 (GraphPad Software, San Diego, CA, USA).

## 3. Results

### 3.1. Isolation and Expansion of MSCs

#### 3.1.1. BM-MSCs

**Figure 2:**
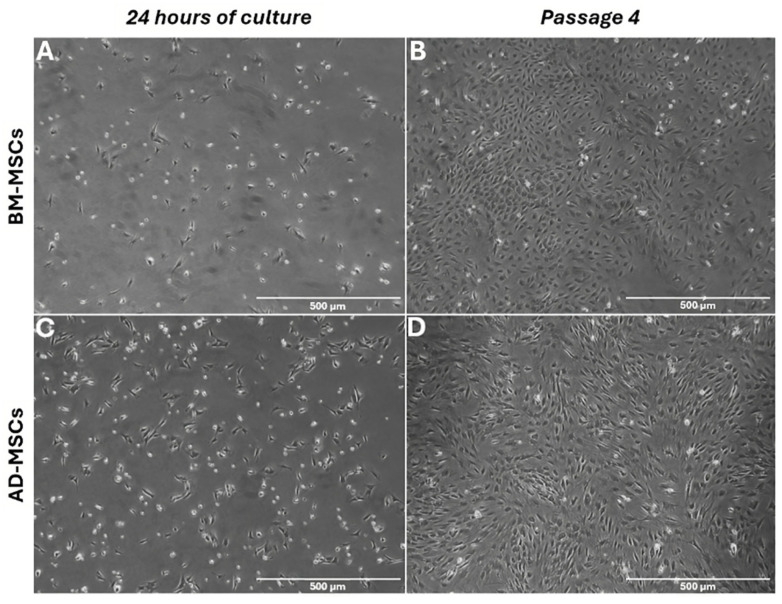
Morphology of plastic-adherent mesenchymal stem cell (MSC) cultures: (**A**) Bone marrow-derived MSCs (BM-MSCs) at day 1 and (**B**) at passage 4. (**C**) Adipose tissue-derived MSCs (AD-MSCs) at day 1 and (**D**) at passage 4. By passage 4, both BM-MSCs and AD-MSCs displayed a uniform population of spindle-shaped cells. The scale bar represents 500 µm.

The primary BM-MSCs initially exhibited a spherical morphology and remained floating in the culture medium. After 24 h, a minimal number of cells adhered to the plastic surface. By the third day, the adhered cells exhibited a circular morphology. With continued culture over several days, these cells gradually assumed a typical spindle-shaped morphology. They reached approximately 80% confluence within 12–14 days of cultivation. Upon subculturing, the cells displayed either a polygonal or elongated spindle-shaped morphology (Figure 2A,B).

#### 3.1.2. AD-MSCs

After 24 h of culture (day 1), the plastic-adherent AD-MSCs primarily exhibited a circular shape. Over time, these cells transitioned into spindle-shaped fibroblast-like cells, which are characteristic of mesenchymal morphology. They proliferated rapidly and achieved 80% confluence within one week (Figure 2C,D).

### 3.2. Surface Marker Expression of MSCs

**Figure 3:**
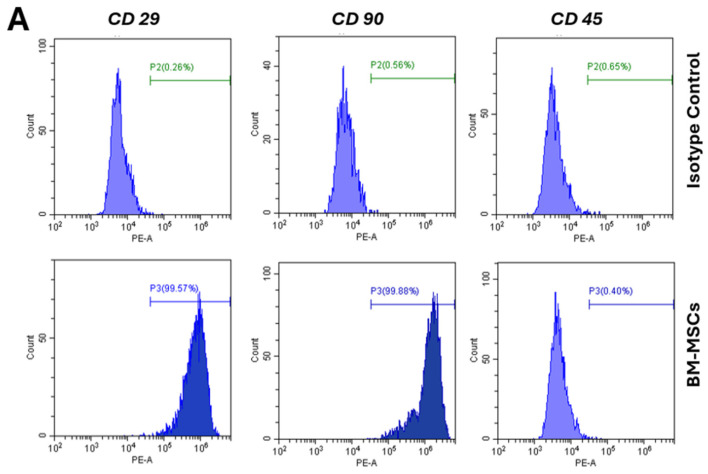

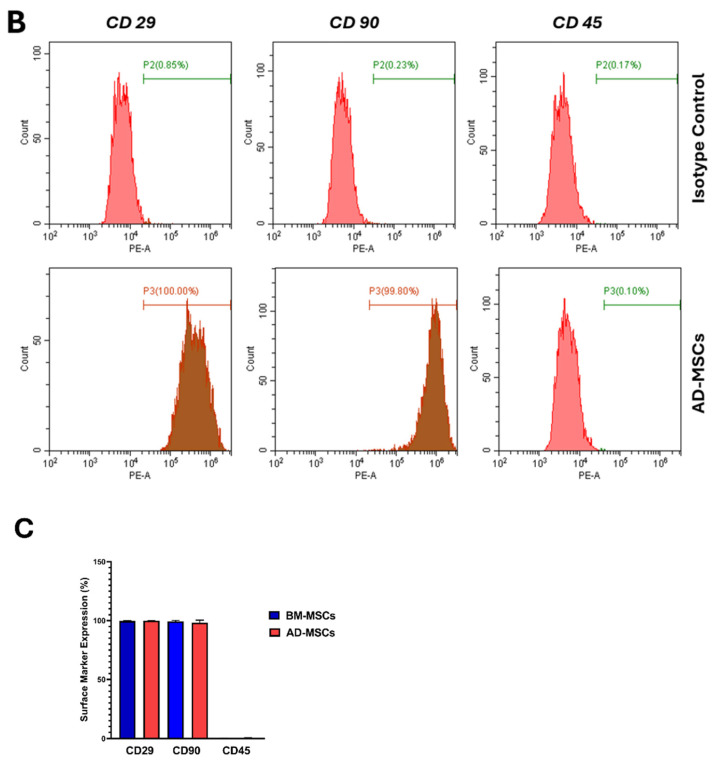
Expression of cell surface markers in passage 4 cells derived from bone marrow and adipose tissue: (**A**,**B**) Representative histograms showing the expression of surface markers: CD29 and CD90 (positive expression), and CD45 (negative expression). (**C**) The percentage values of cell surface markers’ expression are presented. No significant differences were observed in the expression levels of markers between BM-MSCs and AD-MSCs.

Both BM-MSCs and AD-MSCs expressed all the typical MSC surface markers. They exhibited high expression levels of CD29 and CD90, while showing low expression levels of CD45. The representative histograms of surface marker expression are shown in Figure 3A,B. There were no significant differences in the levels of marker expression between BM-MSCs and AD-MSCs: CD29 (99.62 ± 0.19 vs. 99.88 ± 0.10), CD90 (99.34 ± 0.85 vs. 98.17 ± 2.31), and CD45 (0.36 ± 0.12 vs. 0.41 ± 0.29) (Figure 3C). A flow cytometry analysis confirmed the mesenchymal origin of these stem cells.

### 3.3. Differentiation Potential of MSCs

**Figure 4:**
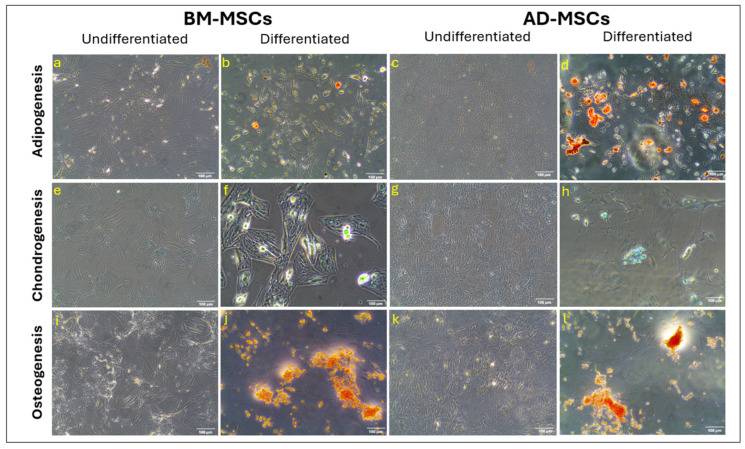
Multi-lineage differentiation of BM-MSCs and AD-MSCs: (i) Adipogenic differentiation is evidenced by positive Oil Red O staining, showing lipid granules in differentiated MSCs (**b**,**d**) compared to undifferentiated cells (**a**,**c**). (ii) Chondrogenic differentiation is confirmed by positive Alcian Blue staining, highlighting highly sulfated proteoglycans in differentiated MSCs (**f**,**h**) versus undifferentiated cells (**e**,**g**). (iii) Osteogenic differentiation is validated by positive Alizarin Red S staining in differentiated MSCs (**j**,**l**) compared to undifferentiated cells (**i**,**k**). The scale bar represents 100 μm.

Both BM-MSCs and AD-MSCs showed successful differentiation into the adipocyte lineage, as indicated by positive Oil Red O staining (Figure 4a–d). The analysis using ImageJ revealed a 5.0-fold increase for AD-MSCs and a 3.7-fold increase for BM-MSCs, suggesting that AD-MSCs have a superior capacity for adipogenic differentiation.

Similarly, both BM-MSCs and AD-MSCs efficiently differentiated into the chondrocyte lineage, as shown by positive Alcian Blue staining (Figure 4e–h). The ImageJ analysis indicated a 3.7-fold increase for BM-MSCs and a 4.7-fold increase for AD-MSCs, suggesting that both cell types have a comparable ability to produce highly sulfated proteoglycans.

In terms of osteogenic differentiation, both BM-MSCs and AD-MSCs were successful, as evidenced by positive Alizarin Red S staining (Figure 4i–l). The ImageJ analysis showed a 4.4-fold increase for BMSCs and a 2.4-fold increase for AD-MSCs, indicating that BM-MSCs have a superior osteogenic differentiation capacity.

### 3.4. Pluripotency and Immunomodulatory Genes

**Figure 5:**
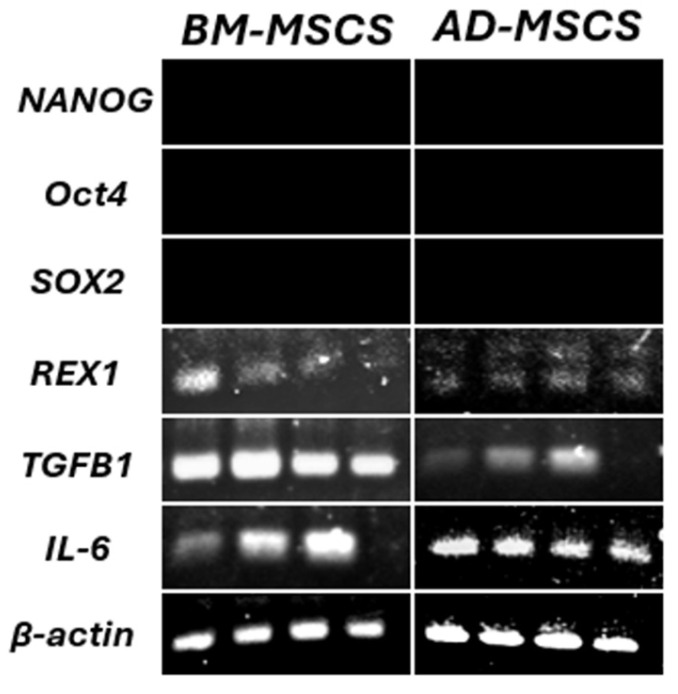
RT-PCR analysis of pluripotency and immunomodulatory gene expression in BM-MSCs and AD-MSCs at passage 4 (*n* = 4): The genes *Oct4*, *Nanog*, and *Sox2* showed negative expression, while *Rex-1*, *TGFB1*, and *IL-6* were positively expressed in both cell types. *β-actin* was used as an internal control.

The findings from the RT-PCR analysis of the pluripotent and immunomodulatory markers, including *Oct4*, *Nanog*, *Sox2*, *Rex-1*, *TGFB1*, and *IL6*, are depicted in Figure 5. Both BM-MSCs and AD-MSCs exhibited no expression of *Oct4*, *Nanog*, and *Sox2*. However, they demonstrated positive expressions of *Rex-1*, *TGFB1*, and *IL6*.

### 3.5. Morphological Alterations

**Figure 6:**
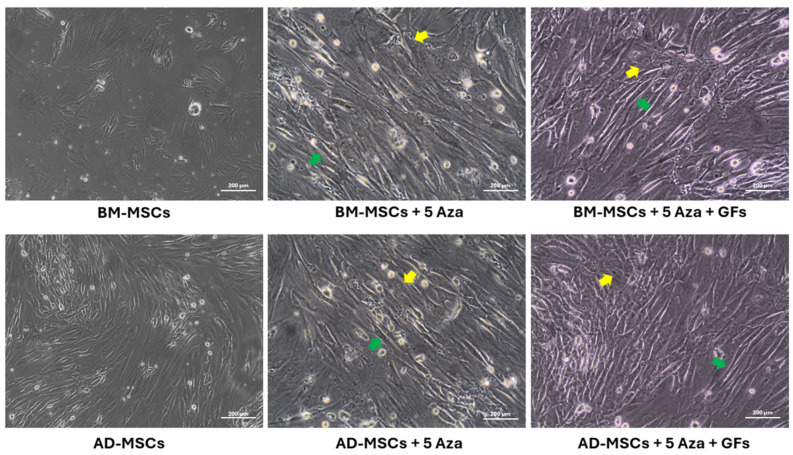
Morphology of BM-MSCs and AD-MSCs after cardiomyogenic differentiation: Yellow arrowheads indicate the appearance of binucleation, while green arrowheads highlight striations and myotube-like arrangements. Scale bar: 200 μm.

After the induction, the culture was monitored daily for any changes in morphology. Within 5 days, an increase in cell size and flattening became apparent. By the third week of induction, the cells extended cytoplasmic projections towards adjacent cells, forming myotube-like structures, a characteristic feature of cardiomyocytes in the induced groups. Notably, there was no observable difference between the two types of cells in this regard. The control cells maintained their spindle shape, with no myotube-like connections observed (Figure 6).

### 3.6. Cell Viability

**Figure 7:**
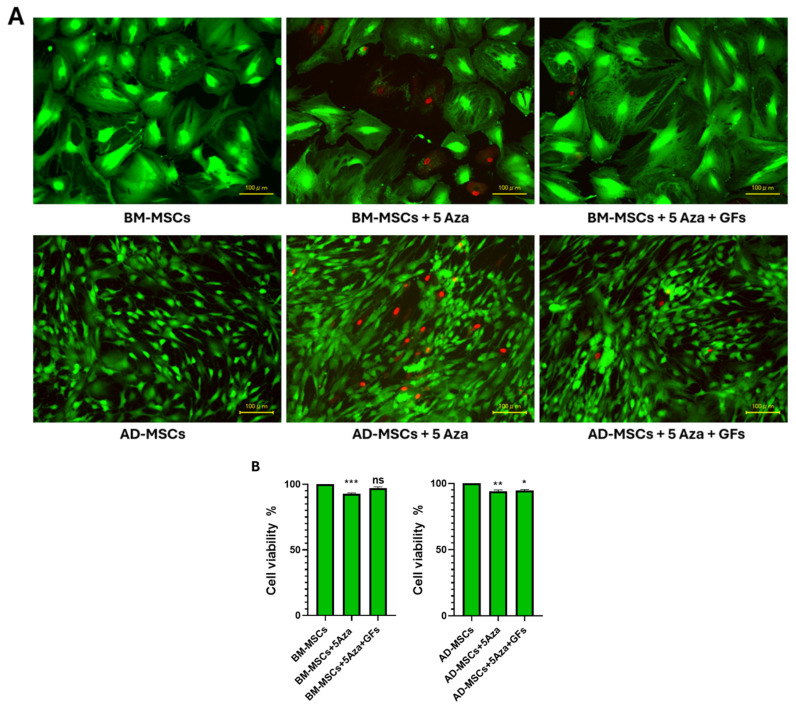
(**A**) Live/dead cell staining of BM-MSCs and AD-MSCs after 7 days of culture in various experimental groups: Living cells are indicated by green fluorescence, while dead cells are indicated by red fluorescence. Scale bar: 100 μm. (**B**) Cell viability was calculated as the percentage of green cells relative to the total number of cells. Statistical significance is indicated as follows: ns (non-significant, *p* > 0.05), * *p* < 0.05, ** *p* < 0.01, and *** *p* < 0.001 compared with the control groups.

All the experimental groups demonstrated a high cell viability, as shown in Figure 7A. However, dead cells positive for the red fluorescent marker were observed in each treated group, with notably higher frequencies seen in the groups treated solely with 5-Azacytidine, particularly in AD-MSCs.

Cell viability was calculated as the percentage of green cells relative to the total number of cells. A significant reduction in cell viability was observed in the (BM-MSCs + 5-Aza) group (92.78%) compared with the control group (BM-MSCs) (*p* = 0.001). In contrast, there were no significant differences between the (BM-MSCs + 5-Aza + GFs) group (97.17%) and the control group (*p* = 0.21). In AD-MSCs, there was a significant reduction in cell viability in the treated groups, either with 5-Aza only (93.97%) or with growth factors (94.53%), compared with the control group (*p* = 0.008 and 0.04, respectively) (Figure 7B).

### 3.7. Expression of Cardiac-Specific Genes

A RT-qPCR analysis was performed to assess the expression of the cardiac-specific markers *MEF2c*, *Troponin I*, and *GSK-3β* among the study groups. *MEF2c* expression was significantly upregulated in BM-MSCs treated with 5-Azacytidine alone and in combination with growth factors, showing increases of 4.8-fold and 10.1-fold, respectively, compared with the untreated BM-MSCs group. Additionally, the combined treatment group exhibited a significantly higher expression compared with the group treated with 5-Azacytidine alone (*p* = 0.0014). Conversely, in AD-MSCs treated with 5-Azacytidine alone and with growth factors, *MEF2c* expression increased by 6.3-fold and 6.9-fold, respectively, compared with the control AD-MSCs, with no significant change between the two treated groups (*p* = 0.99).

*Troponin I* expression in BM-MSCs treated with 5-Azacytidine alone and with growth factors was upregulated by 1.9-fold and 2.7-fold, respectively, compared with untreated BM-MSCs. There was a statistically significant increase in the combined treatment group compared with the 5-Azacytidine alone group (*p* = 0.041). In AD-MSCs treated with 5-Azacytidine alone and with growth factors, *Troponin I* expression increased by 2.7-fold and 4.3-fold, respectively, compared with untreated AD-MSCs, with no significant change between the two treated groups (*p* = 0.095).

Regarding *GSK-3β*, gene expression in BM-MSCs treated with 5-Azacytidine alone and with growth factors was upregulated by 3.9-fold and 2.9-fold, respectively, compared with untreated BM-MSCs, with no significant change between the two treatment groups. In AD-MSCs treated with 5-Azacytidine alone and with growth factors, the upregulation was 4.3-fold and 2.95-fold, respectively, compared with untreated AD-MSCs, again with no significant difference between the treatment groups.

**Figure 8:**
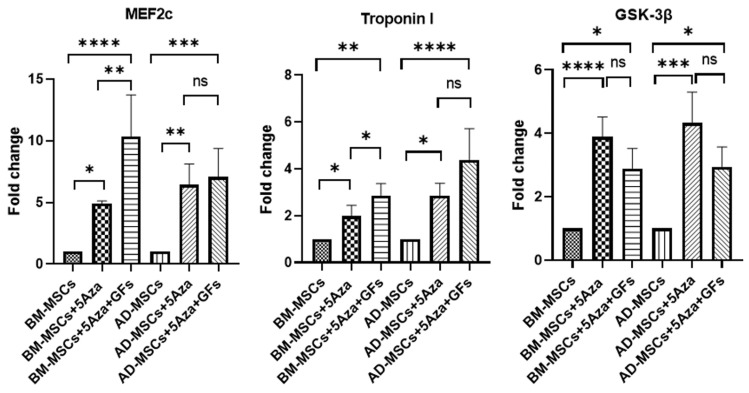
RT-qPCR analysis of gene expression levels in induced BM-MSCs and AD-MSCs: The expression levels of *MEF2c*, *Troponin I*, and *GSK-3β* are shown across different study groups. Statistical significance is indicated as follows: ns, non-significant *p*  >  0.05, * *p*  <  0.05, ** *p*  <  0.01, *** *p*  <  0.001, and **** *p*  <  0.0001.

From these results, we can conclude that BM-MSCs respond more effectively to 5-Azacytidine combined with growth factors than AD-MSCs for the in vitro generation of cardiomyocytes (Figure 8).

### 3.8. Expression of Cardiomyocyte-Specific Proteins

**Figure 9:**
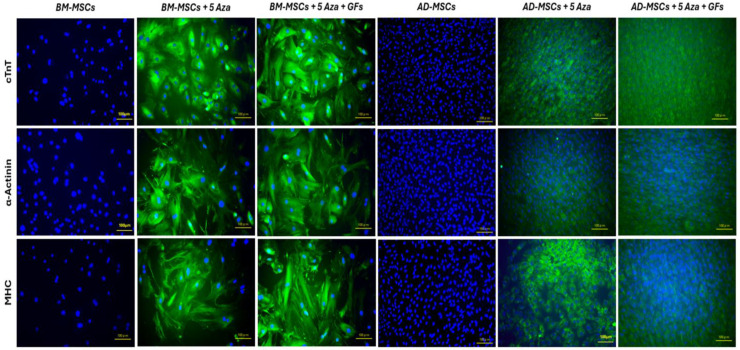
Immunofluorescence staining of cardiomyocyte-specific proteins. Detection of cells positive for Anti-Cardiac Troponin T (cTnT), alpha Actinin 2 (α-Actinin), and Myosin Heavy Chain 1 (MHC) using immunofluorescence microscopy. Scale bar: 100 μm.

To evaluate the differentiation levels of BM-MSCs and AD-MSCs in cardiomyocytes across different study groups, the expression of cardiomyocyte-specific proteins was assessed using immunofluorescence staining. Green fluorescence-labeled cTnT, α-Actinin, and MHC proteins were observed in the cells at the end of the experiment (3 weeks of induction). Both treated groups within each cell type (BM-MSCs and AD-MSCs) expressed these cardiac-specific proteins at comparable levels, with no observable differences between the two treated groups in each cell type. As expected, uninduced MSCs did not exhibit detectable levels of these cardiomyocyte-specific proteins, confirming the specificity of the induction protocol. These findings indicate that both BM-MSCs and AD-MSCs exhibited significant expression of cardiac-specific proteins, suggesting cardiomyogenic differentiation, regardless of the induction protocol used (Figure 9).

## 4. Discussion

In recent decades, MSCs have been extensively researched for their capacity to differentiate into diverse cell types, positioning them as promising candidates in cardiac regenerative therapy [7]. MSCs, multipotent mesenchymal stem cells capable of differentiating into osteocytes, adipocytes, and chondrocytes, possess a strong inclination toward mesodermal lineage differentiation. Studies have consistently demonstrated their capacity to differentiate into cardiomyocytes, highlighting their potential in clinical applications in cardiac therapy [40,41]. Clinical trials addressing CVDs have utilized BM-MSCs and AD-MSCs, yielding promising outcomes. However, studies on a mouse model of MI have demonstrated that preconditioned stem cells exhibit a superior regenerative potential compared with untreated stem cells [42,43].

Our study highlights the benefit of using a low-dose combination of Fibroblast Growth Factor, Insulin-like Growth Factor, and 5-Azacytidine as an effective cardiac inducer in MSCs from both bone marrow and adipose tissue, compared with using 5-Azacytidine alone. In the present study, we utilized freshly isolated BM-MSCs and AD-MSCs. Using the previously reported method [26,27,44], we successfully isolated MSCs from both sources. This process consistently yielded healthy, spindle-shaped cells, which remained viable and proliferated effectively upon replating, resulting in a higher total cell yield.

We observed the morphological changes in the cells daily. The cells adopted a spindle shape after about one day and became more flattened with the increasing passage numbers, consistent with previous studies [26,45]. There were no significant morphological differences between BM-MSCs and AD-MSCs. However, BM-MSCs took longer to reach 70–80% confluence compared with AD-MSCs, which formed multilayers after reaching confluence due to the low contact inhibition, aligning with the results of a previous study [46].

Flow cytometry was used in immunophenotyping characterization, showing no notable distinctions between BM-MSCs and AD-MSCs. Both cell types exhibited positivity for CD29 and CD90, while showing negativity for CD45, which is consistent with the findings reported in previous studies [47,48,49].

The differentiation potential of both BM-MSCs and AD-MSCs was assessed by culturing each cell type in the differentiation-inducing media alongside the control media. Both cell types displayed the capacity to differentiate into osteoblasts, adipocytes, and chondrocytes. However, BM-MSCs exhibited superior osteogenic properties compared with AD-MSCs. Conversely, AD-MSCs demonstrated enhanced adipogenic differentiation, indicated by a higher presence of fat vacuoles in adipocyte-differentiated cells compared with BM-MSCs, aligning with the previous research findings [26,50]. These similarities between BM-MSCs and AD-MSCs [49], combined with the simplicity of adipose tissue extraction, suggest that adipose tissue may represent a more advantageous cell source for tissue repair.

Transcription factors like *NANOG*, *Oct4*, *SOX2*, and *REX1*, renowned for maintaining pluripotency in embryonic stem cells, are also believed to serve analogous functions in adult stem cells. Identifying these pluripotent markers in MSCs is crucial in evaluating their regenerative capacity and overall stem cell attributes [51,52]. The expression of these transcription factors is critical in regulating both self-renewal and differentiation in embryonic stem cells [53,54].

Our study observed that *NANOG*, *Oct4*, and *SOX2* were not expressed in MSCs from either source after four passages, with only *REX1* expression detected. These findings are consistent with the previous research [28], and align with studies showing that *NANOG* is expressed in cultured human adult MSCs, whereas *Oct4*, and *SOX2* are not [54]. However, discrepancies arise when comparing our results with other studies that report the expression of these pluripotency genes in MSCs. For instance, Karaöz et al. [55] found that human bone marrow-derived MSCs expressed *Oct4*, *NANOG*, and *SOX2*, suggesting a potential pluripotent state. Similarly, Greco et al. [56] documented the presence of these embryonic transcription factors in hBM-MSCs, reinforcing the idea that MSCs might retain some pluripotent characteristics. Casella et al. [57] also identified high levels of these stemness markers in rat AD-MSCs.

The differences in pluripotency gene expression between our findings and those of other studies might be attributed to several factors. One critical factor is age-related epigenetic changes, which can impact the expression of pluripotency markers. For instance, histone deacetylation has been shown to influence the expression of *Oct4* and *NANOG* in human embryonic stem cells [58,59]. Moreover, environmental factors, including cell culture conditions, can significantly affect gene expression. The reduced expression of pluripotency markers under normoxic conditions is well-documented and can result in diminished stem cell functionality [60,61].

Recent studies have highlighted the role of extracellular vesicles (EVs) in modulating pluripotency. Jo et al. [62] demonstrated that MSC-derived EVs can influence the self-renewal capacity of MSCs at a genetic level, with old MSCs showing an increased expression of *NANOG* and *Oct4* when cultured with young MSC-derived EVs. This suggests that external factors, such as EVs, can modulate the expression of key transcription factors and contribute to the variability observed in pluripotency gene expression.

Furthermore, Takahashi and Yamanaka [63] highlighted the crucial role of specific transcription factors in regulating pluripotency and differentiation. Their findings suggest that such factors are integral to maintaining stem cell characteristics, which might vary among MSC populations [64]. Additionally, heterogeneity among MSCs, influenced by factors such as donor age and tissue origin, contributes to varying gene expression outcomes. Studies have shown that MSCs exhibit diverse molecular profiles depending on their source and the environmental conditions [65,66,67]. In light of these findings, our study’s results showing the absence of *NANOG*, *Oct4*, and *SOX2* expression can be understood in the context of MSC heterogeneity and the dynamic nature of pluripotency gene expression. This variability in gene expression across different studies may stem from a combination of age-related epigenetic changes, environmental factors, and inherent MSC heterogeneity.

Additionally, in this study, the gene expression of the immunomodulatory markers *TGFB1* and *IL-6* was investigated. *TGFB1*, a multifunctional cytokine, plays a crucial role in diverse cellular processes such as cell growth, differentiation, tissue repair, and immunosuppression [68]. *IL-6*, another multifunctional cytokine, exerts dual roles in the immune system, displaying both pro-inflammatory and anti-inflammatory effects [69].

Our observations revealed that *TGFB1* and *IL-6* were expressed in both cell types, consistent with the findings from previous studies [70,71,72]. In MSCs under normal conditions, their immunomodulatory functions extend to suppressing the proliferation of T cells, influencing the maturation and function of dendritic cells, inhibiting the proliferation and final development of B cells, and adjusting the activities of other immune cells like NK cells and macrophages [73].

The process of inducing cardiac differentiation in BM-MSCs has been thoroughly explored using several inducers, such as 5-Azacytidine, DMSO, and FGF-2. Conversely, studies on cardiac differentiation in AD-MSCs have predominantly focused on MSCs derived from rats and rabbits [74,75]. In this study, we employed 5-Azacytidine (10 μmol) [76] as a cardiac inducer for MSCs, following previous research by Makino et al. [77], which reported that 5-Azacytidine has been shown to induce the differentiation of murine BM-MSCs into cardiomyocytes. Additionally, Antonitsis et al. demonstrated that 5-Azacytidine stimulates the differentiation of MSCs into functional cardiac cells through random DNA demethylation [78]. However, some studies suggest that 5-Azacytidine may lead to the non-specific hypomethylation of cardiac gene promoters, resulting in the generation of non-functional cardiac cells. Moreover, its cytotoxic effects on MSCs pose another challenge [20,79].

To address these limitations, we explored the combination of low doses of growth factors with 5-Azacytidine to enhance their differentiation efficacy towards cardiomyocytes in both cell types. We used optimal concentrations of FGF (1 ng/mL) and IGF (10 ng/mL) to achieve efficient in vitro cardiomyogenic differentiation [31,32].

After treatment with inducers, morphological changes were observed in both BM-MSCs and AD-MSCs in the respective treatment groups. Binucleation and multinucleation were also noted in some cells of both types, possibly linked to the expression of cytoskeletal proteins [80]. The cells exhibited a flattened appearance and adopted a myotube-like morphology, which is characteristic of cardiomyocytes. This was observed in the groups treated with 5-Azacytidine alone and in combination with growth factors [77,80,81].

The Live/Dead assay provided a direct assessment of the proportion of living and dead cells using a two-color fluorescence method. The control groups (BM-MSCs and AD-MSCs) exhibited the fewest dead cells, indicating a higher cell viability under baseline conditions [82,83]. Notably, the (BM-MSCs + Aza + GFs) group showed a relatively lower proportion of dead cells compared with the (AD-MSCs + Aza + GFs) group, suggesting a more favorable response to the combined treatment with growth factors in general, and specifically in BM-MSCs. This may be attributed to the potential effects of FGF and IGF on MSCs. These results align with the findings from Song et al., who reported that MSCs transfected with FGF-2 showed enhanced cell viability and decreased cell death when subjected to hypoxic conditions [84], and Zhang et al., who reported that both IGF-1 and HGF exhibit time-dependent effects by preventing cell death and promoting differentiation into cardiomyocytes in BM-MSCs [85]. Conversely, the groups treated with only 5-Azacytidine displayed a relatively higher proportion of dead cells compared with the other experimental groups, highlighting the potential cytotoxic effects of 5-Azacytidine when used in isolation. These findings are consistent with other studies that have identified the cytotoxic effects of 5-Azacytidine as a major limitation in cardiac regeneration using MSCs [20]. Overall, these findings underscore the effectiveness of combined growth factor treatment in enhancing cell viability during the differentiation process, especially in BM-MSCs.

To delve deeper into the molecular changes during the cardiogenic induction of MSCs, we assessed the expression of structural and functional markers, such as *MEF2c*, *Troponin I*, and *GSK-3β.* These markers are commonly used to demonstrate in vitro cardiac differentiation in MSCs [13]. *MEF2c* functions as a transcriptional regulator involved in cardiovascular development and growth [86] and, as demonstrated by various studies, works in conjunction with *GATA* factors to induce gene transcription in cardiomyocytes [87]. *Troponin I* is a specific cardiac protein involved in forming the contractile network of cardiomyocytes [88] and constitutes an essential component of the cardiac troponin complex, playing a crucial role in maintaining cardiac contraction and relaxation [89]. *GSK-3* is a serine/threonine kinase with diverse functions, including the regulation of hypertrophy and apoptosis in cardiomyocytes. Specifically, *GSK-3β* plays distinct roles in cardiac development in mice [90].

The qPCR results indicated a shift in the mRNA expression of *MEF2c*, *Troponin I*, and *GSK-3β* across all treatment protocols compared with the control group in both cell types. These findings align with studies demonstrating that MSCs pretreated with FGF and hydrocortisone synergistically generated competent cardiomyocytes in vitro compared with MSCs treated with 5-Azacytidine alone [91]. Moreover, this is consistent with another study that showed that pretreating MSCs with IGF-1 resulted in elevated levels of cardiac markers like cTnT, cTnI, and phosphorylated IGF-1. When IGF-1-sensitized MSCs were treated with I-OMe AG538, an inhibitor of phosphorylated IGF-1 receptor kinase, it subsequently decreased the levels of cTnT and cTnI. This suggests that IGF-1 enhances the differentiation of MSCs into cardiomyocytes by increasing the expression of cardiac markers [33]. Conversely, we observed that the expression level of *GSK-3β* was lower in the groups treated with the combination protocol compared with those treated only with 5-Azacytidine in both cell types. These results may be attributed to *GSK-3* being a critical component of the Wnt signaling pathway, which is involved in cell differentiation. Growth factors like IGF and FGF can interact with the Wnt pathway, potentially leading to different regulatory outcomes on *GSK-3* expression. Alternatively, 5-Azacytidine alone might cause more significant demethylation of the *GSK-3* promoter compared with the combination with growth factors, leading to a higher expression. The presence of growth factors could introduce additional layers of regulation that modulate this effect [90].

Notably, our qPCR results demonstrated that BM-MSCs respond more effectively to the combined treatment protocol compared with AD-MSCs, as evidenced by significantly higher expression levels of *MEF2c* and *Troponin I* in the BM-MSC groups treated with both 5-Azacytidine and growth factors, compared with treatment with 5-Azacytidine alone. This enhanced response in BM-MSCs can be attributed to their inherent biological properties and their higher propensity for cardiomyogenic differentiation. Previous studies have highlighted that BM-MSCs possess a greater intrinsic ability to differentiate into cardiomyocytes when stimulated by specific inducers, such as 5-Azacytidine and growth factors, due to their origin and microenvironmental influences in the bone marrow niche [92]. Additionally, the synergistic effect of 5-Azacytidine with growth factors like FGF and IGF has been shown to potentiate the cardiogenic differentiation of BM-MSCs by activating key signaling pathways and transcription factors involved in cardiac lineage commitment [93]. These findings are consistent with our results, suggesting that the combined treatment protocol is more effective in promoting cardiomyocyte-specific gene expression in BM-MSCs, making them a superior cell source for potential therapeutic applications in cardiac regeneration. The lack of a significant difference in AD-MSCs may be attributed to the distinct biological characteristics and the differentiation potential of these cells. Previous research indicates that while AD-MSCs are capable of differentiating into cardiomyocytes, their efficiency and responsiveness to specific induction protocols can vary significantly compared with BM-MSCs [43]. These findings underscore the importance of selecting the appropriate MSC source and optimizing the induction protocols for cardiac regeneration applications.

The immunofluorescence results confirmed the cardiac-specific protein expression of cTnT, α-Actinin, and MHC in all the treatment groups compared with the control groups, consistent with previous studies [43,76]. These results suggest that both 5-Azacytidine alone and in combination with the growth factors used in the study are able to upregulate cardiac-specific proteins. These findings are supported by recent studies showing that more powerful induction factors, such as platelet lysate, can induce BM-MSCs into beating cardiomyocytes [81]. Platelet lysate uniquely serves as a reservoir for both growth factors and differentiation factors [94,95]. Among these factors, FGF is recognized as a proliferative factor that functions via FGF receptors (FGFR1/FGFR2) to support cell cycle progression in cardiomyocyte populations [96]. Similarly, IGF-1 is acknowledged as a factor that induces differentiation, and signaling through the PDGF/PDGFRα interaction activates critical pathways, such as MAP kinase, PLC, and PI3K, which play pivotal roles in cardiomyogenic processes [97].

The results of our study provide promising insights into the potential application of both BM-MSCs and AD-MSCs in regenerative medicine, particularly in cardiac repair. They exhibit significant potential for tissue regeneration due to their ability to differentiate into cardiomyocytes and their capacity for paracrine signaling, which supports tissue repair and reduces inflammation [98,99,100].

Several preclinical studies have demonstrated the efficacy of MSCs in animal models of myocardial infarction, where MSC transplantation led to improved cardiac function and reduced infarct size [101,102]. Translating these findings to human therapy involves addressing several challenges, including optimizing delivery methods, ensuring the survival and integration of transplanted cells, and preventing potential immune rejection [103]. The use of growth factors, as explored in our study, provides a viable strategy to enhance MSC differentiation and survival, thereby improving therapeutic outcomes [104]. Although our study was conducted in rat MSCs, the methodologies and insights demonstrated hold promise for advancing regenerative cardiology and could significantly contribute to the development of innovative treatments. However, further research and clinical trials will be essential to fully realize the potential of MSCs in human regenerative medicine [105,106].

Our study findings indicate that FGF at a dose of 1 ng/mL and IGF at a dose of 10 ng/mL are non-toxic and effective in both BM-MSCs and AD-MSCs. Additionally, BM-MSCs respond more effectively to the combination of these growth factors with 5-Azacytidine than AD-MSCs for the in vitro generation of cardiomyocytes. To our knowledge, this study represents the first comparison of rat BM-MSCs and AD-MSCs regarding their differentiation in the presence of specific growth factors, focusing on both the transcriptional and translational aspects within a unified framework. This initial investigation sets the stage for further exploration into molecular pathways, the interplay of additional structural and functional genes, and the key parameters influencing cardiomyocyte generation. It enhances our comprehension of MSCs’ in vitro differentiation into cardiac cells.

### Limitations

This study primarily investigated the differentiation of bone marrow and adipose tissue mesenchymal stem cells into cardiomyocytes using a singular dose regimen of fibroblast growth factor, insulin-like growth factor, and 5-Azacytidine. While our findings are promising, future research should explore the effects of varying doses of 5-Azacytidine and growth factors to optimize differentiation efficiency and quality. Additionally, the study focused on in vitro experiments, and further research is needed to assess the applicability of these findings in diverse cell sources and in vivo models.

## 5. Conclusions

This study successfully isolated and compared the cardiomyogenic potential of MSCs from bone marrow and adipose tissue in rats. Both BM-MSCs and AD-MSCs can express cardiac-specific markers following induction, suggesting a potential for cardiomyogenic differentiation using 5-Azacytidine, either alone or combined with low doses of FGF and IGF. AD-MSCs showed faster confluence and earlier signs of differentiation. The combined treatment protocol reduced cell mortality and significantly enhanced the expression of cardiac-specific markers in BM-MSCs. These findings suggest that BM-MSCs, particularly when treated with the combined low-dose protocol, may offer a more effective cell source for cardiomyocyte generation and hold greater promise in regenerative medicine applications.

## Figures and Tables

**Table 1:** Specific primers used in RT-PCR.

Purpose	Name	Direction	Primer Sequence
Pluripotent marker genes	*NANOG*	Forward	TACCTCAGCCTCCAGCAGAT
		Reverse	CATTGGTTTTTCTGCCACCT
	*Oct4*	Forward	CGAACCTGGCTAAGCTTCCA
		Reverse	GCCATCCCTCCACAGAACTC
	*SOX2*	Forward	CTCGCAGACCTACATGAAC
		Reverse	TCGGACTTGACCACAGAG
	*REX1*	Forward	GCTCCGGCGGAATCGAGTGG
		Reverse	GCACGTGTTGCTTGGCGACC
Immunomodulatory marker genes	*TGFB1*	Forward	ATGCCAACTTCTGTCTGGGG
		Reverse	GGTTGTAGAGGGCAAGGACC
	*IL6*	Forward	CCACCCACAACAGACCAGTA
		Reverse	TCTGACAGTGCATCATCGCT
Housekeeping gene	*beta-actin*	Forward	CCCATCTATGAGGGTTACGC
		Reverse	TTTAATGTCACGCACGATTTC

**Table 2:** Gene primers used for the RT-qPCR assessment.

Name	Direction	Primer Sequences (5′–3′)
*MEF2c*	Forward	ATGCGGCTCTCTGAAGGATG
	Reverse	TAGCACACACACACACTGCA
*Troponin I*	Forward	GAGCTTCAGGACCTATGCCG
	Reverse	GAGAGTGGGCCGCTTAAACT
*GSK-3β*	Forward	GGATGATGGCCGAGACTCTG
	Reverse	AGGCTCCCTCCAAGATCCAT
*Beta-actin*	Forward	CCCATCTATGAGGGTTACGC
	Reverse	TTTAATGTCACGCACGATTTC

## Data Availability

The data presented in this study are available on request from the corresponding author.
